# Association of Timely Antibiotics and Fluid Resuscitation With Increased Discharge to Home After Sepsis

**DOI:** 10.1016/j.chest.2026.03.002

**Published:** 2026-03-13

**Authors:** Hallie C. Prescott, Julien Weinstein, Sarah Seelye, Jason D. Buxbaum, Steven J. Bernstein, Megan Cahill, Megan Heath, Lama Hsaiky, Jennifer K. Horowitz, Namita Jayaprakash, Anurag N. Malani, Elizabeth McLaughlin, Lakshmi Swaminathan, Patricia J. Posa, Scott A. Flanders, Andrew M. Ryan

**Affiliations:** a Department of Internal Medicine, University of Michigan, Ann Arbor, MI; b Veterans Affairs Center for Clinical Management Research, Ann Arbor, MI; c Veterans Affairs Ann Arbor Healthcare System, Ann Arbor, MI; d Department of Health Services, Policy and Practice, Brown University, Providence, RI; e Henry Ford Macomb Hospital, Clinton Township, Southfield, MI; f Corewell Health System, Southfield, MI; g Henry Ford Hospital, Detroit, MI; h Trinity Health Ann Arbor Hospital, Ann Arbor, MI; i Center for Advancing Health Policy Through Research, Brown University, Providence, RI

**Keywords:** benchmarking, health care quality indicator, hospitalization, risk adjustment

## Abstract

**Background:**

Sepsis is a devastating condition with frequent discharge to nonhome settings such as skilled nursing facilities. Bundled payment incentive programs targeting sepsis have tried to encourage lower spending by avoiding discharge to institutional postacute care.

**Research Question:**

What is the impact of timely antibiotic delivery and fluid resuscitation on discharge to home after sepsis?

**Study Design and Methods:**

This was an observational cohort study of adults hospitalized for confirmed community-onset sepsis at 67 hospitals participating in the Michigan Hospital Medicine Safety Consortium Sepsis Initiative (HMS-Sepsis) from 2022 through 2025. Timely antibiotic delivery and fluid resuscitation were assessed via performance measures used for statewide benchmarking. Antibiotic delivery was measured in patients without positive viral testing results. Target administration was ≤ 3 hours of emergency department arrival among patients with hypotension, or else ≤ 5 hours. Fluid resuscitation (≥ 30 mL/kg body weight) was measured in patients with hypotension or elevated lactate. The primary outcome was discharge to home.

**Results:**

Among 38,568 patients with community-onset sepsis (18,941 male patients [49.1%]; median age, 71 years [interquartile range, 61-80 years]), 7,942 patients (20.6%) died in hospital or were discharged to hospice, 9,941 patients (25.8%) were discharged to a postacute care facility, and 20,685 patients (53.6%) were discharged to home. Among 35,025 and 27,393 eligible patients, timely antibiotic delivery and fluid resuscitation occurred in 26,357 patients (75.3%) and 13,561 patients (49.5%), respectively. In multivariable models adjusted for patient characteristics, timely antibiotic administration and fluid resuscitation were associated with a 3.0-absolute percentage point (95% CI, 2.0-4.0 absolute percentage point) and 1.1-absolute percentage point (95% CI, 0.2-2.1) increase in discharge to home, respectively. Findings were robust across sensitivity and subgroup analyses.

**Interpretation:**

Our results show that in this multihospital cohort, timely antibiotic delivery and fluid resuscitation were associated with increased discharge to home after sepsis. This finding suggests that timely treatment of sepsis may reduce downstream morbidity and health care expenditures.

FOR EDITORIAL COMMENT, SEE PAGE 290

**Take-Home Points**

**Research** **Question:** What is the impact of timely antibiotic delivery and fluid resuscitation on discharge to home after sepsis?

**Results:** In this cohort study of > 35,000 adults hospitalized for confirmed community-onset sepsis, timely antibiotic administration and fluid resuscitation (when indicated) were associated with a 3.0-absolute percentage point (95% CI, 2.0- to 4.0-absolute percentage point) and 1.1-absolute percentage point (95% CI, 0.2- to 2.1-absolute percentage point) increase in discharge to home, respectively, in adjusted analyses.

**Interpretation:** Our results indicate that timely treatment of sepsis is associated with increased discharge to home, suggesting that it may reduce downstream morbidity and health care expenditures.

Sepsis is a devastating condition that results from the immune system’s response to infection.^1^ Each year, in the United States, approximately 1.7 million adults are hospitalized and 270,000 die of sepsis.^2^ In 2018, the costs of hospital care for sepsis totaled > $41 billion for Medicare beneficiaries alone.^3^ Moreover, patients who survive sepsis often experience new morbidity and health impairment,^4^ resulting in high rates of postdischarge health care use, including rehospitalization and discharge to skilled nursing facilities.^5^

Given its high economic and societal burden, sepsis was targeted under Medicare’s Bundled Payment for Care Improvement Advanced program introduced by the Centers for Medicare and Medicaid Services in 2018.^6^ The Bundled Payment for Care Improvement Advanced is a voluntary, bundled payment program that aims to reduce spending and improve patient outcomes by holding hospitals accountable for costs from initial hospitalization, as well as costs of postacute care and rehospitalization occurring within 90 days of hospital admission.^6^ Under this program, hospitals are rewarded financially when health care use and associated costs are lower than expected after discharge.^6^ For sepsis, the bulk of variation in costs of care in the 90 days after admissions is the result of differences in after discharge costs.^5^ These costs vary almost 2-fold across hospitals, driven largely by differences in rehospitalization and discharge to postacute care facilities across hospitals.^5^

Prior research has focused on reducing rehospitalization after sepsis, but less attention has been paid to factors associated with increased ability to discharge to home. In this analysis, we sought to evaluate the impact of early sepsis management on discharge to home. We hypothesized that the cornerstones of sepsis management—timely antibiotic delivery and fluid resuscitation—would be associated with increased discharge to home.

## Study Design and Methods

### Setting and Data Source

The Michigan Hospital Medicine Safety (HMS) Consortium Sepsis Initiative (HMS-Sepsis) is a multihospital collaborative quality initiative funded by Blue Cross Blue Shield of Michigan. HMS-Sepsis aims to improve management and outcomes of sepsis across the state of Michigan. Additional information on HMS and the HMS-Sepsis initiative is provided in e-Appendixes 1 and 2, respectively.

Briefly, professional abstractors at each hospital enter data from a random sample of adults hospitalized for community-onset sepsis into the central HMS-Sepsis registry. Eligible hospitalizations are identified via a 2-step process. First, hospitalizations with a principal diagnosis of sepsis or infection are identified, then abstractors review the electronic health record to confirm presence of infection and acute organ dysfunction during the first 2 calendar days of the encounter. Specifically, abstractors assess for lactate elevation, acute respiratory dysfunction, acutely altered mental status, acute renal dysfunction, acute hematologic dysfunction, acute liver dysfunction, and vasopressor treatment, similar to the Centers for Disease Control and Prevention’s Adult Sepsis Event criteria.^7^ This process ensures a consistent threshold of acute organ dysfunction for inclusion.

Data on eligible hospitalizations, including early management, are entered into the HMS-Sepsis registry using structured abstraction forms and a standardized data dictionary. To ensure robust data collection, abstractors undergo training by the HMS coordinating center and a subset of abstractions are audited for accuracy and completeness.

### Cohort

For this analysis, we included community-onset sepsis hospitalizations at 67 hospitals in the HMS-Sepsis registry discharged from January 1, 2022, through April 7, 2025. We excluded hospitalizations with a principal diagnosis other than sepsis and in which the final discharge location was unknown, including patients transferred to another acute care hospital, those transferred to inpatient psychiatry, or those transferred to a correctional facility.

### Exposures and Outcomes

The exposures of interest were timely antibiotic administration and ≥ 30 mL/kg fluid resuscitation—when indicated—as assessed via HMS-Sepsis performance measures used for statewide benchmarking of sepsis management in Michigan. The inclusion criteria, exclusion criteria, and passing criteria for each process measure are presented in e-Table 1.

Time to antibiotics was measured as the time from emergency department or hospital arrival to time of first administration of a systemic antibiotic included in the Centers for Disease Control and Prevention’s Adult Sepsis Event^7^ surveillance definition. Patients with positive COVID-19 or influenza test results within 2 days of admission were excluded from antibiotic analyses, as were patients without evidence of sepsis on presentation. The Surviving Sepsis Campaign guidelines recommend antibiotic administration within 1 hour of recognition for septic shock and within 3 hours of recognition for sepsis, based on the greater impact of antibiotic delays among patients with shock.^8^, ^9^, ^10^, ^11^, ^12^ HMS-Sepsis considers timely antibiotic delivery to be within 3 hours of hospital arrival for patients with sepsis and hypotension (systolic BP < 90 mm Hg or mean arterial pressure < 65 mm Hg) and within 5 hours of hospital arrival for patients without hypotension, thereby allowing 2 hours for recognition of sepsis. HMS-Sepsis stratifies antibiotic timing targets based on hypotension (not shock) because hypotension is identifiable in real time. By contrast, clinicians do not know which patients will demonstrate shock within 6 to 12 hours. Furthermore, patients with hypotension are at heightened risk for progression to shock, and timely antibiotic initiation decreases this risk.^11^^,^^13^^,^^14^

Fluid resuscitation was assessed in patients with hypotension or lactate elevation, as detailed in e-Table 1. Fluid volumes included resuscitative crystalloid fluids (0.9% normal saline, lactated Ringer’s, or Plasmalyte A: Balanced Salt Solution; Baxter Healthcare Corporation), as well as blood products and albumin. Consistent with the Centers for Medicare and Medicaid Services Severe Sepsis and Septic Shock Early Management Bundle (SEP-1) performance measure,^15^ weight-based resuscitation volume was calculated using actual body weight for patients with a BMI of ≤ 30 kg/m^2^ and ideal body weight for patients with BMI of >30 kg/m^2^.

The HMS-Sepsis registry includes granular data on hospital discharge status. The primary outcome of this study was discharge to home, which included discharge to home with or without home health services, discharge to assisted living with or without home health services, and discharge to custodial nursing care. Discharge to inpatient rehabilitation, long-term acute care, subacute rehabilitation, or skilled nursing were considered discharges to a postacute care facility. Discharges to home hospice, inpatient hospice, or a health care facility with hospice services were considered hospice discharges.

### Primary Analyses

We examined the association of antibiotic timing and fluid resuscitation with discharge to home using a series of logistic regression models, 1 for each process measure assessed. Analyses examining antibiotic timing were adjusted for predicted probability of discharge to home and factors associated with antibiotic timing in a prior HMS-Sepsis analysis^16^: sex, admission from a facility, gastrointestinal symptoms on presentation, reduced left ventricular ejection fraction, and vital signs on presentation. Analyses examining fluid resuscitation were adjusted for predicted probability of discharge to home and multiple factors associated with fluid resuscitation in a prior HMS-Sepsis analysis^17^: age, sex, admission from a facility, hospitalization in the prior 90 days, moderate to severe kidney disease, moderate to severe liver disease, congestive heart failure, malignancy, predicted mortality using HMS-Sepsis Mortality Model,^18^ BMI, initial lactate, initial creatinine, Pao_2_ to Fio_2_ ratio, mechanical ventilation within 6 hours of hospital arrival, vasopressors within 6 hours of hospital arrival, and acutely altered mental status.

Predicted probability of discharge to home was determined via a risk-adjustment model using the same variables and functional forms as the HMS-Sepsis Mortality Model,^18^ but recalibrated for discharge to home. This risk-adjustment model achieved high discrimination, with a C-statistic of 0.845. We present results using adjusted ORs and adjusted difference in discharge to home, but focus on the latter because ORs are not comparable across models.^19^

### Subgroup, Sensitivity, and Exploratory Analyses

We completed multiple subgroup analyses. First, to evaluate the impact of early sepsis management—conditional on survival to hospital discharge—we completed a subgroup analysis of hospital survivors (ie, patients without inpatient mortality or hospice discharge). Second, because discharge to home is an unexpectedly good outcome among patients admitted from a postacute care facility, we completed a subgroup analysis excluding patients admitted from a skilled nursing, subacute rehabilitation, or long-term acute care facility. Third, because our data span several years and were collected in the context of ongoing quality improvement efforts, we completed subgroup analyses in patients admitted during earlier (2022-2023) and later (2024-2025) study years. To assess the robustness of our modeling approach, we completed 3 sensitivity analyses using different approaches to modeling. We additionally examined different weight-based fluid volume cutoffs. To assess for heterogeneity of treatment effect, we completed an exploratory analysis examining sepsis management practices by tertile of predicted probability of 30-day mortality as calculated using the HMS-Sepsis Mortality Model.^18^

Analyses were completed in SAS version 9.4 software (SAS institute). We considered *P* < .05 to indicate statistical significance. This study was deemed not regulated by the University of Michigan institutional review board (Identifier: HUM00188852) because HMS-Sepsis is a quality improvement initiative. Each participating hospital completes a data use agreement governing the use of their HMS-Sepsis registry data.

## Results

### Study Cohort

**Figure 1:**
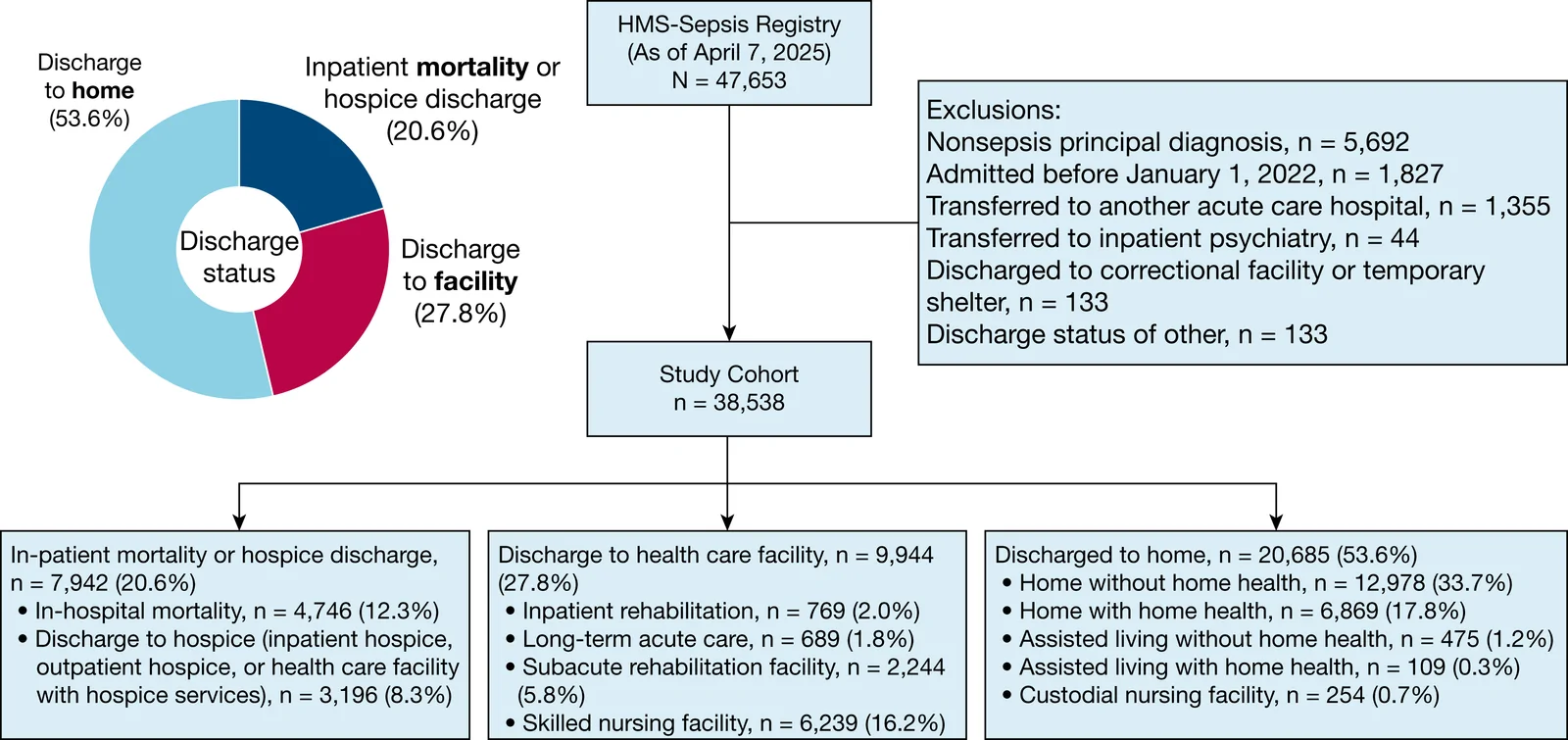
Strengthening the Reporting of Observational Studies in Epidemiology Statement study flowchart. HMS-Sepsis = Michigan Hospital Medicine Safety Consortium Sepsis Initiative.

Among 38,568 patients hospitalized for community-onset sepsis and meeting study criteria, 7,942 patients (20.6%) died in hospital or were discharged to hospice, 9,941 patients (25.8%) were discharged to a postacute care facility, and 20,685 patients (53.6%) were discharged to home. Figure 1 shows the Strengthening the Reporting of Observational Studies in Epidemiology Statement flow diagram.

Baseline patient characteristics are presented in Table 1. The study cohort was 49.1% male, median age was 71 years (interquartile range, 61-80 years), and had a high burden of chronic health conditions, including 39.6% with cardiovascular disease, 40.5% with diabetes, 32.2% with moderate or severe kidney disease, 20.5% with cognitive impairment, and 7.7% with metastatic cancer. In the 90 days before sepsis hospitalization, 34.0% were hospitalized and 15.1% were admitted from a skilled nursing, a subacute rehabilitation, or a long-term acute care facility. Predicted probability of 30-day mortality using the HMS-Sepsis Mortality Model was 0.13 for the median patient (interquartile range, 0.06-0.28). Within 6 hours of presentation, 6.6% of patients were administered invasive mechanical ventilation and 12.2% were administered vasopressor therapy.

**Table 1:** Characteristics of the Study Cohort

Demographic Characteristic	Data
Age, y	71 (61-80)
Male sex	18,941 (49.1)
Recent health care use	
Hospitalized in prior 90 d	13,081 (34.0)
Admitted from SNF, SAR, or LTAC	5,820 (15.1)
Chronic health conditions	
Baseline cognitive impairment	7,890 (20.5)
Baseline functional impairment	0 (0-5)
Cardiovascular disease	15,268 (39.6)
Cerebrovascular disease	6,796 (17.6)
Chronic pulmonary disease	11,962 (31.0)
Dementia	5,451 (14.1)
Diabetes	15,608 (40.5)
Leukemia or lymphoma	1,300 (3.4)
Moderate or severe kidney disease	12,412 (32.2)
Solid malignancy, no metastasis	6,427 (16.7)
Metastatic solid tumor	2,979 (7.7)
Moderate or severe liver disease	1,181 (3.1)
Admission physiologic features	
Maximum creatinine, mg/dL	1.3 (0.9-1.9)
Maximum lactate, mg/dL	2.2 (1.3-3.3)
Acutely altered mental status	19,260 (49.9)
Vasopressors within 6 h	4,707 (12.2)
Mechanical ventilation within 6 h	2,541 (6.6)
Predicted 30-d mortality using HMS-Sepsis Mortality Model	0.13 (0.06-0.28)

Data are presented as No. (%) or median (interquartile range). HMS-Sepsis = Michigan Hospital Medicine Safety Consortium Sepsis Initiative; LTAC = long-term acute care facility; SAR = subacute rehabilitation facility; SNF = skilled nursing facility.

Baseline characteristics differed markedly among patients who died in hospital (or were discharged to hospice), were discharged to a health care facility, and were discharged to home (e-Table 2). For example, median ages were 76 years, 75 years, and 68 years among patients who died, were discharged to a health care facility, or were discharged home, respectively. Predicted probabilities of 30-day mortality were 0.35, 0.17, and 0.07, respectively.

### Prevalence of Timely Antibiotic Administration and Fluid Resuscitation

Of 38,568 patients in the overall cohort, 35,025 patients (90.8%) had no positive test results for COVID-19 or influenza and therefore were eligible for timely antibiotics, of whom 8,460 patients (24.1%) demonstrated hypotension and 26,565 patients (75.8%) did not demonstrate hypotension. Twenty-seven thousand three hundred ninety-three patients (71.0%) were eligible for fluid resuscitation, including 12,176 patients (31.6%) with both a strong clinical indication for fluid resuscitation (ie, hypotension or lactate ≥ 4 mM) and no contraindications to fluid resuscitation (ie, no evidence of end-stage renal disease, reduced left ventricular ejection fraction, or severe to critical aortic stenosis). Among 35,025 and 27,393 eligible patients, timely antibiotic delivery and fluid resuscitation occurred in 26,357 patients (75.3%) and 13,561 patients (49.5%), respectively (e-Table 3). The proportion of eligible patients receiving timely antibiotic delivery and fluid resuscitation increased over the study period (e-Table 4).

### Association of Timely Sepsis Management With Outcomes

**Figure 2:**
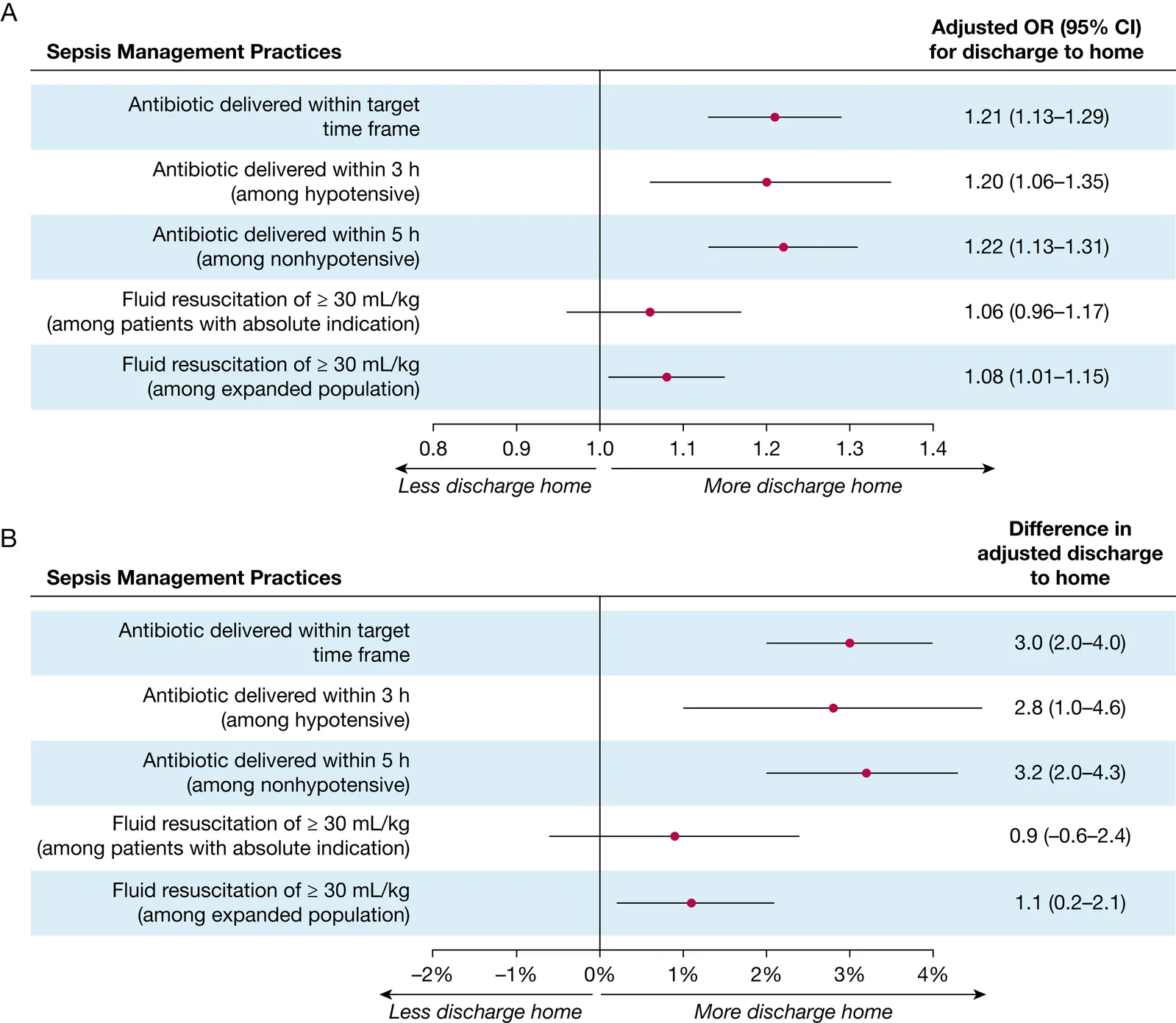
A, B, Forest plots showing the difference in adjusted discharge to home with timely sepsis management: adjusted ORs for discharge to home (A) and differences in adjusted discharge to home (B). Inclusion criteria and definitions for sepsis management practices are shown in e-Table 1. Adjusted differences reflect the absolute differences in adjusted probability of discharge to home (ie, they are on an absolute and not relative scale).

Timely antibiotic delivery (adjusted OR [aOR], 1.21; 95% CI, 1.13-1.29) and fluid resuscitation (aOR, 1.08; 95% CI, 1.01-1.15) were associated with increased odds of discharge to home (Table 2, Fig 2). On an absolute scale, timely antibiotic delivery was associated with a 3.0-percentage point (95% CI, 2.0- to 4.0-percentage point) increase in discharge to home, with similar effect sizes among patients with and without hypotension (Table 2, Fig 2). Fluid resuscitation with ≥ 30 mL/kg was associated with a 1.1-percentage point (95% CI, 0.2- to 2.1-percentage point) increase in discharge to home, with a similar effect size in the subset with both a strong indication and no contraindication to fluid resuscitation (0.9-percentage point increase; 95% CI, –0.6- to 2.4-percentage point).

**Table 2:** Adjusted Outcomes (Discharge to Home) Among Patients Who Received vs Did Not Receive Timely Sepsis Management

Early Sepsis Management Practice	Adjusted Proportion Discharged to Home		Adjusted OR for Discharge to Home (95% CI)	Adjusted Difference in Discharge to Home (95% CI)
	Received the Practice	Did Not Receive the Practice		
Antibiotic delivered within target time frame	54.4 (54.0-54.9)	51.5 (50.6-52.3)	1.21 (1.13-1.29)	3.0 (2.0-4.0)
Antibiotic delivered within 3 h (among hypotensive)	40.1 (39.0-41.1)	37.3 (35.8-38.7)	1.20 (1.06-1.35)	2.8 (1.0-4.6)
Antibiotic delivered within 5 h (among normotensive)	59.0 (58.5-59.6)	55.9 (54.8-56.9)	1.22 (1.13-1.31)	3.2 (2.0-4.3)
Fluid resuscitation of ≥ 30 mL/kg (among patients with absolute indication)	43.1 (42.2-44.0)	42.2 (41.0-43.4)	1.06 (0.96-1.17)	0.9 (-0.6-2.4)
Fluid resuscitation of ≥ 30 mL/kg (among expanded population)	52.0 (51.3-52.7)	50.9 (50.2-51.5)	1.08 (1.01-1.15)	1.1 (0.2-2.1)

Data are presented as % (95% CI) unless otherwise indicated. Inclusion criteria and definitions for sepsis management practices are shown in e-Table 1.

### Subgroup Analyses

Subgroup analyses are presented in Table 3. Among 30,629 survivors of hospitalization, timely antibiotic delivery was associated with increased discharge to home (aOR, 1.18 [95% CI, 1.09-1.27]; adjusted difference, 2.3 [95% CI, 1.2-3.4]), but fluid resuscitation was not (aOR, 0.99 [95% CI, 0.91-1.07]; adjusted difference, –0.2 [95% CI, –1.2 to 0.9]). Among 32,748 patients not admitted from a skilled nursing, subacute rehabilitation, or long-term acute care facility, timely antibiotic delivery (aOR, 1.23 [95% CI, 1.15-1.31]; adjusted difference, 3.7 [95% CI, 2.5-4.8]) and fluid resuscitation (aOR, 1.09 [95% CI, 1.03-1.17]; adjusted difference, 1.6 [95% CI, 0.5-2.8]) were associated with increased discharge to home. Among patients admitted in both earlier study years (2022-2023) and later study years (2024-2025), timely antibiotics and fluid resuscitation were associated with increased discharge to home, with larger effect sizes in the later study years.

**Table 3:** Subgroup Analyses Examining the Association of Timely Sepsis Management and Discharge to Home

Variable	Adjusted OR for Discharge to Home (95% CI)	Adjusted Difference in Discharge to Home (95% CI)
Primary analysis: overall cohort		
Antibiotic delivered within target time frame	1.21 (1.13-1.29)	3.0 (2.0-4.0)
Antibiotic delivered within 3 h (among hypotensive)	1.20 (1.06-1.35)	2.8 (1.0-4.6)
Antibiotic delivered within 5 h (among normotensive)	1.22 (1.13-1.31)	3.2 (2.0-4.3)
Fluid resuscitation of ≥ 30 mL/kg (among patients with absolute indication)	1.06 (0.96-1.17)	0.9 (-0.6-2.4)
Fluid resuscitation of ≥ 30 mL/kg (among expanded population)	1.08 (1.01-1.15)	1.1 (0.2-2.1)
Subgroup 1: hospital survivors (excluding patients who die in the hospital or are discharged to hospice)		
Antibiotic delivered within target time frame	1.18 (1.09-1.27)	2.3 (1.2-3.4)
Antibiotic delivered within 3 h (among hypotensive)	1.14 (0.98-1.32)	2.0 (-0.3-4.3)
Antibiotic delivered within 5 h (among normotensive)	1.20 (1.10-1.31)	2.5 (1.2-3.7)
Fluid resuscitation of ≥ 30 mL/kg (among patients with absolute indication)	0.95 (0.84-1.08)	-0.7 (-2.6-1.1)
Fluid resuscitation of ≥ 30 mL/kg (among expanded population)	0.99 (0.91-1.07)	-0.2 (-1.2-0.9)
Subgroup 2: excluding patients admitted from SNF, SAR, or LTAC facility		
Antibiotic delivered within target time frame	1.23 (1.15-1.31)	3.7 (2.5-4.8)
Antibiotic delivered within 3 h (among hypotensive)	1.22 (1.08-1.38)	3.7 (1.5-6.0)
Antibiotic delivered within 5 h (among normotensive)	1.23 (1.14-1.33)	3.7 (2.4-5.1)
Fluid resuscitation of ≥ 30 mL/kg (among patients with absolute indication)	1.07 (0.97-1.18)	1.3 (-0.5-3.2)
Fluid resuscitation of ≥ 30 mL/kg (among expanded population)	1.09 (1.03-1.17)	1.6 (0.5-2.8)
Subgroup 3: patients admitted in 2022-2023		
Antibiotic delivered within target time frame	1.16 (1.07-1.26)	2.4 (1.1-3.6)
Antibiotic delivered within 3 h (among hypotensive)	1.20 (1.03-1.39)	2.8 (0.4-5.2)
Antibiotic delivered within 5 h (among normotensive)	1.15 (1.04-1.26)	2.2 (0.7-3.7)
Fluid resuscitation of ≥ 30 mL/kg (among patients with absolute indication)	1.05 (0.93-1.20)	0.9 (-1.2-2.9)
Fluid resuscitation of ≥ 30 mL/kg (among expanded population)	1.07 (0.91-1.25)	0.7 (-0.6-2.0)
Subgroup 4: patients admitted in 2024-2025		
Antibiotic delivered within target time frame	1.29 (1.16-1.42)	4.0 (2.4-5.6)
Antibiotic delivered within 3 h (among hypotensive)	1.20 (0.99-1.45)	2.7 (-0.2-5.5)
Antibiotic delivered within 5 h (among normotensive)	1.35 (1.20-1.53)	4.8 (2.9-6.8)
Fluid resuscitation of ≥ 30 mL/kg (among patients with absolute indication)	1.05 (0.96-1.14)	1.0 (-1.4-3.4)
Fluid resuscitation of ≥ 30 mL/kg (among expanded population)	1.12 (1.02-1.24)	1.8 (0.3-3.4)

Inclusion criteria and definitions for sepsis management practices are shown in e-Table 1. Adjusted differences reflect the absolute differences in adjusted probability of discharge to home (ie, they are on an absolute and not relative scale). LTAC = long-term acute care facility; SAR = subacute rehabilitation facility; SNF = skilled nursing facility.

### Sensitivity Analyses

Findings were similar across modelling approaches, as shown in e-Table 5. Findings also were similar when weight-based fluid resuscitation volume was dichotomized at 25 mL/kg or 35 mL/kg, although point estimates were consistently strongest for 25 mL/kg, as shown in e-Table 6.

### Exploratory Analysis of Heterogeneity of Treatment Effect

In an exploratory analysis assessing for heterogeneity of treatment effect, timely antibiotic initiation was associated with increased discharge to home across all 3 tertiles of predicted 30-day mortality, with larger effect sizes in the middle tertile of risk (e-Table 7, e-Fig 1). Timely fluid resuscitation also was associated increased discharge home across tertiles of risk, but the difference in adjusted discharge to home was not significant in medium- or highest-risk patients (e-Table 7, e-Fig 1).

## Discussion

In this multihospital cohort of > 35,000 patients hospitalized for confirmed community-onset sepsis, timely antibiotic delivery and fluid resuscitation were associated with increased discharge to home. Conditional on hospital survival, timely antibiotic initiation, but not fluid resuscitation, remained associated with increased discharge to home. Overall, these findings suggest that timely sepsis management not only improves survival, but also increases discharge to home.

Timely antibiotic delivery and fluid resuscitation are cornerstones of sepsis management because they improve short-term survival.^8^ They also are presumed to improve longer-term, nonmortality outcomes,^4^ although improved short-term survival does not always translate to improved nonmortality outcomes.^20^ Indeed, experts have cautioned that reductions in short-term mortality may have no impact on survival with debility, or alternatively, may have a morbidity or mortality tradeoff whereby more patients survive with debility.^20^ Antibiotic therapy, although indicated for treatment of all bacterial infections causing sepsis,^21^ may cause antibiotic-associated adverse events, including allergic reactions, medication toxicities, development of *Clostridioides difficile* infection, and increased antibiotic resistance.^22^ Antibiotics with antianaerobic coverage may be particularly problematic because of their depleting effects on the gut microbiome,^23^^,^^24^ so should be avoided outside of specific indications for anaerobic coverage.^21^ If more prompt antibiotic delivery is associated with differences in antibiotic selection and spectrum of coverage, it is plausible that a morbidity and mortality tradeoff could occur with shorter time to antibiotic delivery.^9^ Reassuringly, however, this study suggests that, in a large, multihospital cohort of patients with confirmed sepsis receiving real-world care, timely antibiotic delivery was associated with increased discharge to home. This finding is consistent with the 2018 Prehospital Antibiotics Against Sepsis (PHANTASi) trial of prehospital antibiotics for sepsis, where the intervention arm received antibiotics a median of 96 minutes faster than the control arm.^25^ Patients randomized to the intervention arm showed similar short-term mortality (relative risk of 0.95, which was not statistically significant given the sample size and relatively low illness severity of the trial cohort), but a lower rate of 28-day rehospitalization (7% vs 10%; *P* = .004), suggesting a potential benefit of timely antibiotic delivery on longer-term recovery.^25^^,^^26^ Like timely antibiotic delivery, fluid resuscitation is recommended because of its impact on short-term survival.^8^ However, also like antibiotics, potential harms can result from too much fluid, although typically these occur with resuscitation volumes far exceeding 30 mL/kg.^21^ In 1 observational cohort of patients treated for septic shock, 86% showed a positive fluid balance and 35% were volume-overloaded at discharge from the ICU.^26^ Volume overload at ICU discharge was associated independently with inability to walk at hospital discharge and new discharge to a health care facility,^26^ suggesting another potential morbidity and mortality tradeoff and underscoring the need to consider active de-resuscitation in the recovery phase of septic shock. Our study suggests that, overall, fluid resuscitation with ≥ 30 mL is associated with increased discharge to home but, conditional on survival, has no impact on discharge to home. Furthermore, our exploratory analysis of heterogeneity of treatment effect suggested that the benefits of fluid resuscitation on discharge to home were greatest among lower-risk patients. We hypothesize that fluid resuscitation may increase discharge home in lower-risk patients, whereas it may lead to increased survival, but discharge to postacute care facilities, in higher-risk patients.

This finding suggests the need for further research to inform personalization of fluid resuscitation and de-resuscitation, which is an area of increased attention.^27^ Guidelines suggest use of diuretics for fluid removal after the acute phase of fluid resuscitation,^27^ but use in real-world practice is variable.^26^^,^^28^

Our study was motivated by the Centers for Medicare and Medicaid Services Bundled Payment for Care Improvement Advanced program, which incentivizes hospitals to reduce rehospitalization and facility discharge after sepsis. Although many hospitals trying to reduce costs in the postdischarge period may focus on peridischarge care coordination and avoidance of facility discharge in lower-risk patients, our study suggests that focusing on early sepsis management also may improve longer-term nonmortality outcomes and recovery.

Our study should be considered in the context of several limitations. First, like all observational studies, this study is at risk of residual confounding, which could bias findings in either direction. However, we leveraged granular clinical registry data that facilitate risk-adjustment models with high discrimination.^18^ Second, we evaluated timely management using dichotomous performance measures. However, antibiotic timing is a continuous measure, without a clear threshold effect, whereas fluid resuscitation may vary in volume, timing, and composition. Nevertheless, thresholds are needed for performance benchmarking. We used robust criteria for assessing timely management that are informed by the best available evidence and are in operational use for statewide benchmarking in Michigan. Third, we focused specifically on antibiotic timing and fluid volume, which have been the focus of recent performance improvement in sepsis, but other aspects of early management (eg, antibiotic selection, fluid composition) influence outcomes. Further work is needed to evaluate the impact of other aspects of treatment on discharge to home. Fourth, we were unable to assess the appropriateness of discharge to home. We considered discharge to home to be a favorable outcome because it is preferred by most patients and is less costly. However, some patients discharged to home may have benefitted from discharge to a facility. Likewise, we were unable to evaluate the initial recommended discharge location, only the actual discharge disposition. Fifth, our study focused on patients with confirmed community-onset sepsis. We are unable to measure the impact of timely management on the broader population of patients treated for potential sepsis (ie, inclusive of patients with noninfectious conditions mimicking sepsis). The benefit of timely treatment for confirmed sepsis coupled with the potential harms of unnecessary antibiotic therapy and fluid resuscitation highlights the need for urgent evaluation to clarify diagnosis in patients with potential sepsis. Finally, although patients with positive test results for COVID-19 or influenza were excluded from antibiotic timing measures, patients with culture-negative sepsis (some of whom might have had sepsis as a result of other viral infections^29^) were included, which may bias the results toward the null. Strengths of the study include the large sample size, the recency of the data, the robustness of findings across sensitivity and subgroup analyses, and the diversity of hospitals included, ranging from large urban academic medical centers to smaller rural hospitals.

## Interpretation

In this multihospital cohort, timely antibiotic delivery and fluid resuscitation were associated with increased discharge to home after sepsis. Conditional on survival, timely antibiotic delivery remained associated with increased discharge to home, suggesting that timely antibiotic delivery not only improves survival, but also reduces downstream morbidity resulting from sepsis. Fluid resuscitation was associated with improved discharge to home, but showed no difference in discharge to home conditional on hospital survival.

## Funding/Support

This work was supported by Blue Cross Blue Shield of Michigan as part of their Value Partnerships program and by the National Heart, Lung, and Blood Institute [Grant R01HL160588 (A. R.)]. This work was also supported with resources and use of facilities at the Ann Arbor VA Medical Center.

## Financial/Nonfinancial Disclosures

The authors have reported to *CHEST* the following: H. C. P., J. W., S. J. B., M. H., J. K. H., P. J. P., E. M., S. A. F.) receive salary support from Blue Cross Blue Shield of Michigan for work on HMS. The authors report grant funding and salary support unrelated to this study from the National Institutes of Health (H. C. P.), Agency for Healthcare Research and Quality (H. C. P.), the Department of Veterans Affairs (H. C. P., S. J. B.), and the Centers for Disease Control and Prevention (H. C. P., J. K. H.). H. C. P. serves on the Surviving Sepsis Campaign guidelines committee and is a paid scientific consultant to Aurobac Therapeutics. None declared (S. S., J. D. B., M. C., L. H., N. J., A. N. M., L. S., A. M. R.).

## Supplementary Data

e-Online Data

e-Online Data
