# Supplementary material for: Association of Timely Antibiotics and Fluid Resuscitation With Increased Discharge to Home After Sepsis
Source: Chest. 2026 Mar 13;170(2):410–9. doi: 10.1016/j.chest.2026.03.002 (PMC13494420; doi:10.1016/j.chest.2026.03.002)
Supplement: Supplementary file 1 [file mmc1.docx]

**ONLINE SUPPLEMENT to** Optimal early sepsis management is associated with increased discharge to home

Table of Contents

[e-Appendix 1: Michigan Hospital Medicine Safety (HMS) Consortium Information	2](#_Toc223784838)

[e-Appendix 2: HMS-Sepsis Registry	5](#_Toc223784839)

[eTable 1: Definitions for performance measures assessing early sepsis management practices	7](#_Toc223784840)

[eTable 3: Receipt of recommended early sepsis management practices	9](#_Toc223784841)

[eTable 4: Receipt of recommended early sepsis management practices by year of admission	10](#_Toc223784842)

[eTable 5: Sensitivity analysis using different modeling approaches	11](#_Toc223784843)

[eTable 6: Sensitivity analysis using different weight-based fluid resuscitation volumes	12](#_Toc223784844)

[eTable 7: Exploratory analysis of heterogeneity of treatment effect by tertile of predicted 30-day mortality	13](#_Toc223784845)

[eFigure 1: Exploratory analysis of heterogeneity of treatment effect by tertile of predicted 30-day mortality	14](#_Toc223784846)

# **e-Appendix 1**: Michigan Hospital Medicine Safety (HMS) Consortium Information

Locations of HMS hospitals.

**General Overview**

The Michigan Hospital Medicine Safety Consortium (HMS) is a Collaborative Quality Initiative (CQI), launched in 2010 with 16 participating hospitals. The goal of HMS is to improve the quality of care for hospitalized medical patients who are at risk for adverse events. In 2025, HMS membership includes 67 hospital partners across the state of Michigan

The HMS CQI targets hospitalists, as well as intensivists and emergency medicine physicians. Due to their management of diverse groups of hospitalized patients and close interactions with hospital staff such as sub-specialists, nurses, and pharmacists, hospitalists are ideally positioned to champion quality improvement (QI) efforts with a goal of preventing adverse events in hospitalized patients.

**Sponsorship/Overall Funding Model**

HMS is one of more than 20 CQIs sponsored by Blue Cross Blue Shield of Michigan (BCBSM) as a part of their [Value Partnerships Program](https://www.bcbsm.mibluedaily.com/value-partnerships). Value Partnerships is a collection of programs from BCBSM, physicians, and hospitals across Michigan that make health care work better for everyone. It's a unique, collaborative model using data collection and sharing of best practices to help health professionals improve patient outcomes.

Value Partnerships improve the quality of care, making it more cost-effective based on the following guiding principles:

- Partnering with providers to deliver clinical programs and aligned provider payments that improve the delivery of high quality, affordable health care
- Designing and executing programs in a customized and collaborative manner, rather than using a one-size-fits-all approach
- Offering a portfolio of interdependent programs that collectively drive outcomes
- Focusing on investments in transformation of care processes
- Recognizing and rewarding the performance of organizations as well as individual providers
- Rewarding improvement to create meaningful incentives for all participants and partner organizations
- Encouraging collaboration among participants

**Pay for Performance**

BCBSM’s Hospital Pay-for-Performance (P4P) program recognizes short term acute-care hospitals for achievements and improvements in quality and population-health management. Specifically related to the CQIs, hospitals can earn up to 40% of their P4P points based on performance. To determine hospital-specific performance, each CQI annually develops a Hospital Performance Index Scorecard to assess a hospitals performance over a given year related to key quality initiatives.

For more detailed information, please visit the BCBSM Value Partnership [website](https://www.bcbsm.com/providers/network/value-partnerships/).

**HMS Leadership & Committees**

The work of the collaborative is led by a leadership team within the HMS Coordinating Center and two committees: the executive committee and the data, design, and publication (DDP) committee.

*HMS Coordinating Center*

Michigan Medicine houses the Coordinating Center for HMS and is responsible for collecting and analyzing comprehensive clinical data from the participating hospitals. It uses these analyses to evaluate and understand current practice, support the implementation of improvement strategies, and to evaluate change over time. The Coordinating Center supports participants in establishing quality improvement goals and assists them in implementing best practices. For additional Coordinating Center details, please visit the HMS (<https://www.mi-hms.org/>).

*Executive Committee*

The purpose of the executive committee is to determine the mission and vision of the collaborative, review current performance and address consortium operational issues, identify and discuss implementation strategies, and establish the future course. The executive committee meets once per year – usually in the Fall – in person. The committee members consist of Coordinating Center Leadership, BCBSM leadership, implementation scientists/local quality experts, and leaders from member hospitals.

*Data, Design, and Publications (DDP) Committee*

The purpose of the HMS Data, Design, and Publications (DDP) Committee is to review and approve data collection methods, performance index measures, programming issues, data analysis methods, quality improvement materials, and the scholarly work of the collaborative. Physician Champions and Quality Improvement Leads/Administrators from all hospitals are invited to participate on this Committee. A clinical data abstractor lead also participates in this Committee as a representative for that role in discussions. The DDP meets monthly via teleconference and 10 to 15 members attend regularly.

*Critical Care Steering Committee*

The purpose of the Critical Care Steering Committee is to help drive improvement efforts in intensive care units across Michigan. Engage, support, and disseminate HMS critical care-focused scholarly work, and identify opportunities for collaboration across disciplines caring for the hospitalized medical patient (i.e., general medicine, emergency medicine, infectious diseases, pharmacy, vascular access, etc.).

**Consortium Building/Recruitment**

Eligible hospitals within the State of Michigan are recruited annually. In 2020, HMS became one of seven required CQIs within the BCBSM Value Partnerships portfolio. If a hospital is eligible for participation in a required CQI and, at the time of recruitment (when the coordinating center reaches out to ask a hospital to join), voluntarily elects not to participate, the hospital forfeits the ability to earn the P4P incentive attributed to the CQI. HMS welcomes approximately 10 hospitals to the consortium each year. The below diagram shows the hospital onboarding since the inception of the collaborative. For a complete list of participating hospitals, please the [HMS website](https://www.mi-hms.org/).

**Hospital Participation - Eligibility and Expectations**

To participate in HMS, a hospital must meet the following eligibility criteria:

- Be enrolled in BCBSM’s Participating Hospital Agreement.
- Have a physician/physician group dedicated to the care of patients in the inpatient hospital setting focused on the care of general medical patients including critical care. For example, hospital program, general internist group, etc.
- Have a sufficient inpatient medical discharge volume of at least 25 eligible adult patients (age 18 years or older) per quarter. Eligibility is based on current QI initiatives. All patients do not have to be managed by the hospitalist program.
- Have organizational commitment for active participation.
- Have an onsite physician champion(s) to act as Clinical Champion.
- Engage members of the multidisciplinary team as it relates to collaborative initiatives. For example, infectious diseases (ID), pharmacy, vascular access, interventional radiology, infection prevention, critical care, etc.
- Have a QI/administrative lead who will work on the collaborative initiatives.
- Have or hire a clinical data abstractor (many of the medical record based data elements to be collected and the follow-up patient calls will require someone with a clinical / healthcare background). While the Collaborative doesn’t require a nurse for the role of the clinical data abstractor, it is highly recommended. More than 95% of the clinical data abstractors are nurses.
- Participate in all initiatives in which the hospital is eligible for participation

**Additional Information**

For more information about HMS, please visit the HMS website (http://mi-hms.org/) or follow us on X (@HMS_MI), Bluesky (@hms-mi.bsky.social) or LinkedIn (<https://www.linkedin.com/company/mi-hms>). For information related to the CQIs, please visit www.valuepartnerships.com.

# **e-Appendix 2:** HMS-Sepsis Registry

Data collection for the HMS-Sepsis registry involves the abstraction of patient data for 18 eligible cases every 2-week abstraction cycle.

Why we require 18 hospitalizations per abstraction period. Hospitals are required to abstract 18 cases per two-week period. Blue Cross Blue Shield of Michigan funds 1 FTE data abstractor at each hospital. We complete time trials each summer to determine the average time to complete data abstractions. We use these data to set abstraction requirements after accounting for vacation and other duties (e.g. participation in collaborative-wide meetings, completion of biannual survey, time for quality improvement activities, etc.).

How hospitals determine which hospitalizations to abstraction. Potentially eligible hospitalizations during the abstraction period are identified at each hospital based on standardized criteria provided to the hospitals (e.g., diagnostic codes, age). Potentially eligible hospitalizations identified by the standardized criteria are then sorted by minute of discharge time-stamp (i.e., a pseudo-random number). Abstractors then review potentially eligible hospitalizations in order until 18 eligible hospitalizations have been identified and abstracted. Approximately one-third of hospitalizations reviewed are ineligible (generally due to lack of acute organ dysfunction on encounter day 1 or 2). Data are entered into the HMS-Sepsis registry via a HIPAA-compliant website that has decision support embedded. The website will indicate whether hospitalizations are eligible vs ineligible after initial data are entered, after which abstractors complete the abstraction for eligible hospitalizations or move to the next case in their list. Compliance with this process is audited by the coordinating center to ensure that hospitals are abstracting a random sample of sepsis hospitalizations.

Inclusion criteria (all 3 required)

- Principal discharge diagnosis of sepsis, pneumonia, influenza, COVID-19, or respiratory failure (with secondary diagnosis of pneumonia, influenza, or COVID)
- Receipt of qualifying antimicrobial within 2 calendar days of presentation, from CDC’s adult sepsis event definition, unless primary diagnosis of COVID
- Acute organ dysfunction present within 2 calendar days of presentation. For principal discharge diagnosis of pneumonia, influenza, or COVID, there must be acute respiratory dysfunction.
  - Supplemental oxygen >4L (35% FIO_2_) for >2 hours on a given calendar day and no history of chronic respiratory failure (J96.11) or chronic home oxygen
  - Serum Cr >1.2 AND ≥50% increase from baseline (lowest value during hospitalization)

AND no end-stage renal dysfunction (N18.6) or stage V chronic kidney disease prior to admission.

- Platelet count <100 cells/μL AND > 50% decline in platelets from baseline (highest value during hospitalization)
- Total bilirubin ≥ 2.0 mg/dL AND doubling of total bilirubin from baseline (lowest value

during hospitalization)

- Lactate > 2.0 mmol/L
- Treatment with intravenous vasopressor (Angiotensin II, Dopamine, Epinephrine,

Norepinephrine, Phenylephrine, or Vasopressin) outside of operating room.

- Clinical documentation of altered mental status on presentation

INCLUDE: Confusion, lethargy, reports that the patient is acting out of usual character,

worsening deviation from baseline mental status, somnolence, comatose state,

encephalopathy, unresponsiveness.

Exclusion criteria

- Pregnant patient
- Patient aged <18 years
- Patient transferred from another hospital (ED to ED Transfer is eligible)
- Major surgery within 2 calendar days of admission
- Patient received IV or IM antibiotics in the 24 hours prior to hospital encounter start
- Comfort measure only / hospice within 3 hours of presentation
- Patient left against medical advice (AMA) or refused medical care
- Length of hospitalization >120 days
- Hospitalization is within 90 days of a prior hospitalization by the same patient that is already abstracted into the HMS-Sepsis registry
- Primary discharge diagnosis is COVID, but there is no positive testing for COVID
- Patients with principal diagnosis of pneumonia, influenza, or COVID who are on chronic supplemental oxygen prior to admission

| **eTable 1:** Definitions for performance measures assessing early sepsis management practices | | | |
| --- | --- | --- | --- |
| **Performance measure** | **Inclusion criteria** | **Exclusion criteria** | **Passing** |
| Timely antibiotic delivery (all patients) | All HMS-sepsis hospitalizations. | 1. Positive COVID-19 or influenza testing on presentation 2. Principal discharge diagnosis of COVID-19 or influenza 3. No symptoms of sepsis on presentation (< 2 SIRS criteria, serum lactate < 2.0 mmol/L, WBC 4,000-12,000, no provider documentation of infectious symptoms) | Administration of a systemic (i.e., intravenous or enteral) antimicrobial included in the CDC Adult Sepsis Event surveillance definition within 3 hours of arrival to hospital or emergency department (if hypotensive), else within 5 hours |
| Timely antibiotic delivery (among hypotensive patient) | HMS-Sepsis hospitalizations with hypotension within 2 hours of presentation. Hypotension is identified by SBP < 90 mm Hg, MAP < 65 mm Hg, or initiation of intravenous vasopressor therapy. |  |  |
| Timely antibiotic delivery (among normotensive patients) | HMS-Sepsis hospitalizations without hypotension during the first 2 hours of presentation. |  |  |
| Fluid resuscitation (hypo-perfused, no contraindications) | HMS-Sepsis hospitalizations with hypoperfusion within 3 hours of presentation. Hypoperfusion is identified by SBP < 90 mm Hg, MAP < 65 mm Hg, initiation of intravenous vasopressor therapy, or serum lactate ≥ 4mmol/L. | 1. Reduced left ventricular ejection fraction (i.e., LVEF < 40%) 2. Stage V chronic kidney disease or end-stage renal disease 3. Severe or critical aortic stenosis | Administration of ≥ 30ml/kg fluid within 6 hours of presentation.  Qualifying fluid includes resuscitative crystalloids (0.9% normal saline, lactated Ringer’s, and PlasmaLyte), blood products, and albumin. Weight-based fluid volume is determined using actual body weight for patients with a body mass index ≤ 30 kg/m^2^ and ideal body weight for patients with a body mass index > 30 kg/m^2^. |
| Fluid resuscitation (expanded population) | HMS-Sepsis hospitalizations with hypoperfusion or intermediate lactate value within 3 hours of presentation. Hypoperfusion is identified by SBP < 90 mm Hg, MAP < 65 mm Hg, initiation of intravenous vasopressor therapy, or serum lactate ≥ 2 mmol/L. | No exclusions for patients with hypoperfusion (as identified SBP < 90 mm Hg, MAP < 65 mm Hg, initiation of intravenous vasopressor therapy, or serum lactate ≥ 4mmol/L).  For patients with intermediate lactate elevation only (lactate ≥ 2 mmol/L to <4), the following exclusions:   4. Reduced left ventricular ejection fraction (i.e., LVEF < 40%) 5. Stage V or end-stage renal disease   Severe or critical aortic stenosis |  |

| **eTable 2**: Patient characteristics by discharge status | | | |
| --- | --- | --- | --- |
|  | In-hospital mortality or hospice discharge,  N=7,942 | Discharge to healthcare facility,  N=9,941 | Discharge to home,  N=20,685 |
| Age in years, median (Q1-Q3) | 76 (67-84) | 75 (66-82) | 68 (57-77) |
| Male sex, N (%) | 3,748 (47.2) | 4,885 (49.1) | 10,308 (49.8) |
| Hospitalized in prior 90 days | 3,701 (46.7) | 3,863 (38.93) | 5,517 (26.8) |
| Admitted from SNF, SAR, LTAC | 2,017 (25.4) | 3,550 (35.7) | 253 (1.2) |
| Chronic health conditions |  |  |  |
| Baseline cognitive impairment | 2,312 (29.2) | 3,018 (30.4) | 2,560 (12.4) |
| Baseline functional impairment | 0 (0-5) | 0 (0-5) | 0 (0-1) |
| Cardiovascular disease | 3,549 (44.7) | 4,284 (43.1) | 7,435 (35.9) |
| Cerebrovascular disease | 1,588 (20.0) | 2,328 (23.4) | 2,880 (13.9) |
| Chronic pulmonary disease | 2,604 (32.8) | 3,114 (31.3) | 6,244 (30.2) |
| Dementia | 1,732 (21.8) | 2,071 (20.8) | 1,648 (8.0) |
| Diabetes | 2,884 (36.3) | 4,490 (45.2) | 8,234 (39.8) |
| Leukemia or lymphoma | 337 (4.4) | 260 (2.6) | 703 (3.4) |
| Moderate or severe kidney disease | 2,984 (37.6) | 3,677 (37.0) | 5,751 (27.8) |
| Solid malignancy, no metastasis | 1,475 (18.6) | 1,597 (16.1) | 3,355 (16.2) |
| Metastatic solid tumor | 1,235 (15.6) | 473 (4.8) | 1,271 (6.1) |
| Moderate or severe liver disease | 444 (6.0) | 220 (2.2) | 517 (2.5) |
| Admission physiology |  |  |  |
| Maximum serum creatinine, mg/dL | 1.50 (1.0-2.5) | 1.30 (1.0-2.1) | 1.17 (0.9-1.7) |
| Maximum serum lactate, mg/dL* | 2.40 (1.2-4.1) | 2.20 (1.3-3.3) | 2.2 (1.3-3.1) |
| Acutely altered mental status | 5,600 (70.5) | 6,211 (62.5) | 7,449 (36.0) |
| Vasopressors within 6 hours | 2,066 (26.0) | 1,257 (12.6) | 1,384 (6.7) |
| Mechanical ventilation within 6 hours | 1,313 (16.5) | 700 (7.0) | 528 (2.6) |
| Predicted 30-day mortality using HMS-Sepsis Mortality Model, med (Q1-Q3) | 0.35 (0.19-0.56) | 0.17 (0.09-0.30) | 0.07 (0.03-0.15) |
| Data are presented as No. (%) or median (interquartile range) unless otherwise indicated. Baseline functional impairment was defined as dependency on others for completion of any of the following activities or instrumental activities of daily living: bathing, dressing, toileting, transferring between bed and chair, walking, and managing medications. | | | |

| **eTable 3**: Receipt of recommended early sepsis management practices | | | | |  |
| --- | --- | --- | --- | --- | --- |
| Early sepsis management practice | Received, N | Eligible,  N | Received, % | Predicted 30-day mortality using HMS-Sepsis Mortality Model, median (IQR) | |
|  |  |  |  | Received | Not Received |
| Antibiotic delivered within target time frame | 26,357 | 35,025 | 75.3 | 0.13 (0.05-0.28) | 0.13 (0.06-0.27) |
| Antibiotic delivered within 3 hours (among hypotensive) | 5,710 | 8,460 | 67.5 | 0.27 (0.13-0.51) | 0.23 (0.11-0.44) |
| Antibiotic delivered within 5 hours (among normotensive) | 20,647 | 26,565 | 77.7 | 0.10 (0.05-0.22) | 0.10 (0.05-0.21) |
| Fluid resuscitation of ≥ 30ml/kg (among patients with absolute indication) | 7,885 | 12,176 | 64.7 | 0.22 (0.10-0.44) | 0.23 (0.11-0.43) |
| Fluid resuscitation of ≥ 30ml/kg (among expanded population) | 13,561 | 27,393 | 49.5 | 0.16 (0.06-0.35) | 0.14 (0.06-0.29) |

| **eTable 4:** Receipt of recommended early sepsis management practices by year of admission | | | | |
| --- | --- | --- | --- | --- |
|  | % receiving (received / eligible) | | | |
| **Overall Cohort** | 2022 | 2023 | 2024 | 2025 |
| Antibiotic delivered within target time frame | 73.3  (4598/6273) | 74.3  (10931/14705) | 77.0  (10578/13734) | 79.9  (250/313) |
| Antibiotic delivered within 3 hours (among hypotensive) | 64.7  (995/1537) | 66.8  (2308/3456) | 69.3  (2355/3397) | 74.3  (52/70) |
| Antibiotic delivered within 5 hours (among normotensive) | 76.1  (3603/4736) | 76.7  (8623/11249) | 79.6  (8223/10337) | 81.5  (198/243) |
| Fluid resuscitation of ≥ 30ml/kg (among patients with absolute indication) | 64.7  (1452/2245) | 64.2  (3191/4973) | 65.4  (3167/4845) | 66.4  (75/113) |
| Fluid resuscitation of ≥ 30ml/kg (among expanded population) | 50.0  (2438/4880) | 48.6  (5526/11368) | 50.3  (5461/10863) | 48.2  (136/282) |
| **Hospital survivors** |  |  |  |  |
| Antibiotic delivered within target time frame | 73.5  (3582/4873) | 74.9  (8550/11422) | 77.8  (8245/10605) | 78.8  (190/241) |
| *Antibiotic delivered within 3 hours (among hypotensive) | 64.9  (652/1005) | 66.0  (1481/2244) | 69.1  (1516/2195) | 74.3  (26/35) |
| Antibiotic delivered within 5 hours (among normotensive) | 75.8  (2930/3868) | 77.0  (7069/9178) | 80.0  (6729/8410) | 79.6  (164/206) |
| Fluid resuscitation of ≥ 30ml/kg (among patients with absolute indication) | 63.7  (967/1519) | 65.4  (2214/3388) | 66.4  (2190/3298) | 65.7  (46/70) |
| Fluid resuscitation of ≥ 30ml/kg (among expanded population) | 48.7  (1771/3634) | 48.3  (4121/8529) | 49.9  (4066/8146) | 46.2  (97/210) |

| **eTable 5**: Sensitivity analysis using different modeling approaches | | | | |
| --- | --- | --- | --- | --- |
| Early sepsis management practice | aOR (95% CI) for association of care practice with discharge to home | | | |
|  | Primary  analysis* | Sensitivity analysis 1* | Sensitivity analysis 2* | Sensitivity analysis 3* |
| Antibiotic delivered within target time frame | 1.21 (1.13-1.29) | 1.19 (1.12-1.27) | 1.17 (1.10-1.24) | 1.17 (1.10-1.24) |
| Antibiotic delivered within 3 hours (among hypotensive) | 1.20 (1.06-1.35) | 1.15 (1.03-1.29) | 1.13 (1.01-1.27) | 1.13 (1.01-1.27) |
| Antibiotic delivered within 5 hours (among normotensive) | 1.22 (1.13-1.31) | 1.22 (1.13-1.31) | 1.20 (1.11-1.28) | 1.19 (1.11-1.28) |
| Fluid resuscitation of ≥ 30ml/kg (in pts with abs. indication) | 1.06 (0.96-1.17) | 1.08 (0.99-1.19) | 1.08 (0.98-1.18) | 1.08 (0.98-1.18) |
| Fluid resuscitation of ≥ 30ml/kg (in expanded population) | 1.08 (1.01-1.15) | 1.08 (1.02-1.15) | 1.08 (1.01-1.15) | 1.08 (1.01-1.15) |
| The primary analysis was a fully-adjusted single-level logistic regression model. The first sensitivity analysis was a single-level logistic regression model adjusted for only predicted probability of discharge to home. The second sensitivity analysis was a logistic mixed effect model with treating hospital as a random effect and adjustment for predicted probability of discharge to home. The third sensitivity analysis was a logistic mixed effect model with treating hospital as a random effect, adjustment for predicted probability of discharge to home, and adjustment for several hospital characteristics: hospital size (<250; 250-499, 500+ beds); Urbanicity using RUCC (metro [RUCC 1-3] vs non-metro [RUCC 4-9]); teaching status (teaching vs non-teaching); and system status (part of system vs stand-alone).Hospitals with fewer than 25 observations were merged to a single cluster for sensitivity analyses 2 and 3. | | | | |

| **eTable 6**: Sensitivity analysis using different weight-based fluid resuscitation volumes | | |
| --- | --- | --- |
| Early sepsis management practice | Adjusted odds ratio for discharge to home (95% CI) | Adjusted difference in discharge to home  (95% CI) |
| **Sensitivity analysis**: Fluid resuscitation of ≥ 25 ml/kg (among patients with absolute indication) | 1.13 (1.02-1.25) | 2.0 (0.3-3.6) |
| **Primary analysis**: Fluid resuscitation of ≥ 30ml/kg (among patients with absolute indication) | 1.06 (0.96-1.17) | 0.9 (-0.6-2.4) |
| **Sensitivity analysis**: Fluid resuscitation of ≥ 35 ml/kg (among patients with absolute indication) | 1.02 (0.93-1.12) | 0.3 (-1.2-1.8) |
| **Sensitivity analysis**: Fluid resuscitation of ≥ 25 ml/kg (among expanded population) | 1.14 (1.06-1.21) | 2.0 (1.0-3.0) |
| **Primary analysis**: Fluid resuscitation of ≥ 30ml/kg (among expanded population) | 1.08 (1.01-1.15) | 1.1 (0.2-2.1) |
| **Sensitivity analysis**: Fluid resuscitation of ≥ 35 ml/kg (among expanded population) | 1.06 (0.99-1.13) | 0.9 (-0.2-1.9) |
| Inclusion criteria and definitions for sepsis management practices are shown in **eTable 1**. Adjusted differences reflect the absolute differences in adjusted probability of discharge to home (*i.e.*, they are on an absolute and not relative scale). | | |

| **eTable 7**: Exploratory analysis of heterogeneity of treatment effect by tertile of predicted 30-day mortality | | | |
| --- | --- | --- | --- |
| Early sepsis management practice | Predicted probability  of discharge to home (95% CI) | | Adjusted difference in discharge to home  (95% CI) |
|  | Received | Not Received |  |
| **Tertile 1 (Lowest risk: bottom third of predicted 30-day mortality*)** | | | |
| Antibiotic delivered within target time frame | 81.7 (81.0-82.4) | 80.1 (78.8-81.4) | 1.6 (0.1-3.1) |
| Antibiotic delivered within 3 hours (among hypotensive) | 78.6 (76.1-81.1) | 76.5 (73.0-80.0) | 2.1 (-2.3-6.5) |
| Antibiotic delivered within 5 hours (among normotensive) | 82.0 (81.3-82.8) | 80.5 (79.1-81.9) | 1.5 (-0.1-3.1) |
| Fluid resuscitation of ≥ 30ml/kg (among patients with absolute indication) | 80.2 (78.4-82.1) | 78.2 (75.6-80.8) | 2.0 (-1.2-5.3) |
| Fluid resuscitation of ≥ 30ml/kg (among expanded population) | 82.3 (81.2-83.4) | 80.6 (79.6-81.7) | 1.6 (0.1-3.2) |
| **Tertile 2 (Medium risk)** | | | |
| Antibiotic delivered within target time frame | 54.6 (53.7-55.6) | 50.1 (48.4-51.7) | 4.5 (2.6-6.5) |
| Antibiotic delivered within 3 hours (among hypotensive) | 55.6 (53.4-57.8) | 50.7 (47.7-53.8) | 4.9 (1.0-8.7) |
| Antibiotic delivered within 5 hours (among normotensive) | 54.4 (53.4-55.5) | 49.7 (47.7-51.7) | 4.7 (2.5-7.0) |
| Fluid resuscitation of ≥ 30ml/kg (among patients with absolute indication) | 55 (53.2-56.8) | 52.9 (50.4-55.4) | 2.1 (-1.1-5.2) |
| Fluid resuscitation of ≥ 30ml/kg (among expanded population) | 54.9 (53.4-56.3) | 53.4 (52.1-54.7) | 1.4 (-0.6-3.4) |
| **Tertile 3 (Highest risk: top third of predicted 30-day mortality*)** | | | |
| Antibiotic delivered within target time frame | 26.4 (25.6-27.2) | 23.5 (22.0-25.9) | 2.9 (1.3-4.6) |
| Antibiotic delivered within 3 hours (among hypotensive) | 22.3 (21.0-23.6) | 20.3 (18.4-22.1) | 2.1 (-0.2-4.3) |
| Antibiotic delivered within 5 hours (among normotensive) | 29.3 (28.1-30.4) | 25.7 (23.6-27.7) | 3.6 (1.2-6.0) |
| Fluid resuscitation of ≥ 30ml/kg (among patients with absolute indication) | 22.6 (21.4-23.9) | 23.1 (21.5-24.7) | -0.5 (-2.5-1.6) |
| Fluid resuscitation of ≥ 30ml/kg (among expanded population) | 24.6 (23.5-25.7) | 24.3 (23.2-25.4) | 0.3 (-1.3-1.9) |
| Inclusion criteria and definitions for sepsis management practices are shown in **eTable 1**. Adjusted differences reflect the absolute differences in adjusted probability of discharge to home (*i.e.*, they are on an absolute and not relative scale).Predicted 30-day mortality was calculated using the validated HMS-Sepsis mortality model^1^ which predicted mortality based on demographics, chronic health, and acute physiologic data from the first 6 hours of presentation. | | | |
|  | | | |

# **eFigure 1**: Exploratory analysis of heterogeneity of treatment effect by tertile of predicted 30-day mortality

Inclusion criteria and definitions for sepsis management practices are shown in **eTable 1**. Adjusted differences reflect the absolute differences in adjusted probability of discharge to home (*i.e.*, they are on an absolute and not relative scale).

1.	Prescott HC, Heath M, Munroe ES, et al. Development and Validation of the Hospital Medicine Safety Sepsis Initiative Mortality Model. *Chest.* 2024;166(5):1035–1045.
